# Popular knowledge of stroke in São Paulo: a cross-sectional study within the World Stroke Campaign

**DOI:** 10.1590/1516-3180.2020.0116.r1.18112020

**Published:** 2021-03-12

**Authors:** Marina Trombin Marques, Mila Carvalho Guachala, Vinícius Andreoli Schoeps, Marcel Simis, Manoel Carlos Sampaio de Almeida Ribeiro, Rubens José Gagliardi

**Affiliations:** I MD. Resident Physician, Discipline of Neurology, Department of Medicine, Irmandade da Santa Casa de Misericórdia de São Paulo, São Paulo (SP), Brazil.; II MD. Resident Physician, Department of Surgery, Irmandade da Santa Casa de Misericórdia de São Paulo, São Paulo (SP), Brazil.; III MD. Physician, Discipline of Neurology, Department of Medicine, Irmandade da Santa Casa de Misericórdia de São Paulo, São Paulo (SP), Brazil.; IV MD, MSc, PhD. Professor, Neuromodulation Laboratory, Institute of Physical Medicine and Rehabilitation, Hospital das Clínicas (HC), Universidade de São Paulo (USP), São Paulo (SP), Brazil.; V MD, PhD. Professor, Discipline of Preventive Medicine, Department of Collective Health, Irmandade da Santa Casa de Misericórdia de São Paulo, São Paulo (SP), Brazil.; VI MD, PhD. Full Professor, Discipline of Neurology, Department of Medicine, Irmandade da Santa Casa de Misericórdia de São Paulo, São Paulo (SP), Brazil.

**Keywords:** Cerebrovascular disorders, Primary prevention, Stroke, Surveys and questionnaires, Cerebrovascular disease, Stroke knowledge, Stroke prevention, Stroke detection

## Abstract

**BACKGROUND:**

Stroke is the second leading cause of death in Brazil and the main cause of disability. Inability to identify alarm signals causes delays in seeking emergency services, thereby leading to a worse prognosis.

**OBJECTIVES:**

To assess the population's knowledge of how to recognize and prevent stroke.

**DESIGN AND SETTING:**

Prospective cross-sectional study on data derived from a questionnaire that was administered during the 2016 World Stroke Campaign, launched in the city of São Paulo, Brazil.

**METHODS:**

Data on 806 interviewees were evaluated using descriptive statistics and univariate and multivariate analyses.

**RESULTS:**

Among all the interviewees, 52.1% knew how to conceptualize stroke; 70.07% knew someone who had suffered a stroke; and 29.03% listed three or more risk factors. Only 27.5% mentioned controlling high blood pressure as a preventive measure. In the event of witnessing a stroke, 57.8% would call the emergency service and 2.9% would check the timing. Less educated individuals were 5.6 times more likely (95% confidence interval, CI 3.45-9.02) to have poor knowledge of stroke, compared with the more educated group. Knowing someone who had had a stroke reduced the chances of not knowing the terms relating to the disease (odds ratio, OR = 0.56; 95% CI 0.4-0.78).

**CONCLUSIONS:**

Despite the severity and prevalence of stroke, the population still has little information on this disease. In this context, the importance of mounting campaigns to improve prevention and treatment and to contribute to healthcare policies becomes evident.

## INTRODUCTION

Stroke is the second most impactful condition that leads to mortality, accounting for 5.7 million deaths worldwide in 2016.^1^^,^^2^ According to the Health Informatics Department of the Brazilian Ministry of Health, stroke is also the second leading cause of mortality in Brazil, accounting for 102,965 deaths in 2016,^3^ and is the leading cause of disability among adults.^1^ According to a national home-based epidemiological survey on health conducted in Brazil, an estimated 2,223,000 people had suffered a stroke and 568,000 had been left with a severe disability (absolute figures). The prevalence was higher among individuals of more advanced age, those with less education and those living in urban areas.^4^

Among the factors leading to worse outcomes from stroke in Brazil is a lack knowledge of the disease. This causes delays in seeking medical care and impairs prevention, thereby resulting in a worse prognosis.^5^

The neurology team at the institution where the present study was developed has joined forces with the World Stroke Campaign. Through this partnership, an awareness campaign on stroke has been under development since 2000. The key points emphasized within this awareness campaign have been how to prevent, identify and react to stroke through knowing its warning signs.

## OBJECTIVE

To assess the population's knowledge of how to prevent and recognize stroke.

## METHOD

This was a prospective cross-sectional study on data from a questionnaire administered within the context of the World Stroke Campaign in Brazil, on October 29, 2016.

The sample evaluated was composed of random passers-by assessed within the campaign who voluntarily showed interest in participating. People who were less than 18 years of age were excluded from the sample. Demographically, the interviewees were characterized according to their gender, age group and education level, and according to the interview site.

The passers-by were asked whether they knew something about stroke, firstly using the term “acidente vascular cerebral” (AVC) and then using the most popular colloquial term used in our region, “derrame”. People were asked what the symptoms of stroke were, and what could be done to avoid stroke. Afterwards, the interviewer would explain the definition and symptoms for people who had not answered the first question correctly. They were then asked what they would do if they identified an acute onset of those symptoms. The interviewer explained the importance of seeking medical help quickly and introduced the concept of stroke as a medical emergency, with a thrombolytic time window and high levels of morbimortality. After the interviewer had explained the potential disabilities relating to stroke, he asked whether the interviewees knew anyone who had suffered a stroke. The questions were open and responses were not induced. **Table 1** presents the questions and the answers that were considered correct.

**Table 1:** Questionnaire

Open question	Answers considered correct
What is stroke (in Portuguese, acidente vascular cerebral)? What is “derrame”?	Interruption of blood circulation in the brain or encephalic bleeding “A brain infarction”
How do you identify a person *who is having* a stroke? What are the symptoms?	Weakness Mental confusion or aphasia Visual impairment Dizziness or coordination impairment Paresthesia Sudden intense headache
How can strokes be avoided?	Hypertension control Dyslipidemia control Physical activity Healthy diet Obesity treatment Avoidance of smoking, excessive drinking and stress
What should you do if you identify someone *who is having* a stroke?	Seek immediate medical attention (by calling 192, which is the emergency number in Brazil) Pay attention to the time of symptom onset

The questionnaire was administered by students enrolled at 14 medical schools in the state of São Paulo. A training course focusing on the main risk factors, preventive measures and key signs and symptoms of stroke had previously been provided by professors to these students. The students were divided into groups, which were then sent to four different subway stations (República, Sé, Barra Funda and Tatuapé) and a public square (Parque da Água Branca), which are in different zones of the city of São Paulo, during one Saturday (October 29, 2016).

This survey had previously been granted approval by the Research Ethics Committee of Irmandade da Santa Casa de Misericórdia de São Paulo, São Paulo (SP), Brazil, under Certificate of Submission for Ethical Appraisal number 60023316.9.0000.5479, on September 16, 2016.

The data were analyzed using descriptive statistics and univariate and multivariate analyses. The univariate analysis characterized interviewees as either knowing or not knowing three or more symptoms, risk factors and protective factors. To evaluate lack of knowledge about stroke in the multivariate analysis, the interviewees were assessed on whether or not they knew the Portuguese-language terms for “stroke” (acidente vascular cerebral or “derrame”).

The multivariate analysis was performed by means of logistic regression, using the stepwise forward strategy to construct the model and considering P = 0.05 for variable input and P = 0.10 for keeping a variable in the model.

## RESULTS

A total of 825 people were interviewed and 806 questionnaires were analyzed (19 needed to be excluded because they had been poorly filled out or the interviewee's age was less than 18 years).

The demographic profile is shown in **Table 2**. In this population, 70.07% knew someone who had had a stroke.

**Table 2:** Demographic profile of the population sample, compared with data for the state of São Paulo

		n = 806 (%)		State of São Paulo
**Gender** *				
	Male	390	49%	47.9%
	Female	413	51%	52.1%
**Age groups, in years**				
	20-39	170**	21%**	35.7%
	40-59	315	39%	25.6%
	60-79	294	36%	06.5%
	> 79	23	3%	01.4%
**Level of education**				
	Incomplete elementary school	135	17%	41.9%
	Completed elementary school	151	19%	18.8%
	Completed high school	316	39%	27.5%
	Completed tertiary education	203	25%	11.0%

* Three individuals were excluded from this analysis due to data missing from their questionnaires

** Four individuals were excluded from this analysis because they were between 18 and 19 years of age, since there are no data relating to the state of São Paulo for comparison.

With regard to knowledge of stroke, 47.9% did not know how to define stroke or “derrame”. This trend was seen to decrease with increasing education level in a statistically significant manner: from 73.48% in the least educated group to 32.23% in the group that had completed tertiary education. In a stratified analysis, those who knew someone who had suffered a stroke showed statistically significant better knowledge of the disease (P = 0.001) (**Table 3**).

**Table 3:** Univariate analysis on not knowing the meaning of the Portuguese-language terms for stroke (“AVC” or “derrame”) and on calling an emergency service when a stroke is identified

Age group (years)		Did not know the meaning of either term					
		20-59		60 and over		Total	
		Freq	%	Freq	%	Freq	%
**Gender**							
	Female	110	43.1	77	48.7	187	45.3
	Male	108	46.6	99	62.7	197	50.5
**Level of education**							
	Incomplete elementary school	54	**76.1** *	43	**70.5** *	97	**73.5** *
	Completed elementary school	52	**58.4**	39	**65.0**	91	**61.1**
	Completed high school	77	**37.7**	53	**48.2**	130	**41.4**
	Completed tertiary education	34	**28.3**	31	**37.3**	65	**32.0**
**Do you know anyone who had suffered a stroke?**							
	No	76	**56.7** *	49	59.0	125	**57.6** *
	Yes	144	**40.6**	117	50.0	261	**44.3**

Age group (years)		Would call an emergency service					
		20-59		60 and over		Total	
		Freq	%	Freq	%	Freq	%
**Gender**							
	Female	155	60.8	100	63.3*	255	61.7*
	Male	128	55.2	81	51.3	209	53.6
**Level of education**							
	Incomplete elementary school	28	**39.4** *	23	**37.7** *	51	**38.6** *
	Completed elementary school	47	**52.8**	31	**51.7**	78	**52.3**
	Completed high school	128	**62.7**	75	**68.2**	203	**64.6**
	Completed tertiary education	80	**66.7**	51	**61.4**	131	**64.5**
**Do you know anyone who has suffered a stroke?**							
	No	68	**50.7** *	42	50.6	110	**50.7** *
	Yes	216	**60.8**	140	59.8	356	**60.4**

* P < 0.05; Freq = frequency.

Regarding the other questions, 29.03% listed three or more risk factors, 18.1% listed three or more symptoms and 67.8% cited at least one preventive measure; 57.8% would call the emergency service, but only 2.9% would check the timing of onset of the signs and symptoms of stroke. The most frequently mentioned symptom was paresthesia (33.6%), followed by dizziness/motor coordination impairment (24.06%), weakness (23.8%), headache (23.2%), mental confusion/aphasia (19.4%) and visual impairment (7.3%).

Among the risk factors, the one that was most cited was obesity (48.2%), followed by sedentary lifestyle (40.5%), systemic arterial hypertension (27.5%), smoking (16.3%), alcoholism (14.01%), dyslipidemia (12.15%) and diabetes mellitus (8%). Among all the interviewees, 29.03% listed three or more risk factors. In a stratified analysis according to education level, this rate rose to 34.19% among those with the highest education level, while among those with the lowest education level it was only 7.69%. This difference was statistically significant (P = 0.01).

The preventive measure most frequently mentioned by the interviewees was avoidance of obesity (48.51%), followed by physical activity (40.5%), high blood pressure control (27.5%), smoking cessation (16.5%), avoidance of alcoholism (14.01%), controlling dyslipidemia (12.15%) and control over diabetes mellitus (8%). Altogether, 25.06% of the interviewees mentioned three or more preventive measures, with a statistically significant difference (P < 0.05) in the analysis stratified according to education level: 32.67% of the individuals who had completed tertiary education and 7.93% of those with incomplete elementary education.

When participants were asked how they would proceed if they witnessed a stroke episode, 57.8% responded that they would call the emergency service, while only 2.9% would check the timing of onset of signs and symptoms and 2.3% would do both. Among this minority that would proceed correctly, 84.2% knew someone who had had a stroke (**Table 3**).

Accepting P < 0.05 as significant, a multivariate analysis was performed to investigate the relationship between the variables and lack of knowledge about stroke.

Education level was a statistically significant factor, since the group with the lowest education level (incomplete elementary school) was 6.1 times more likely (95% confidence interval, CI 3.73-9.96) to have poor knowledge, compared with the group with the highest education level (completed tertiary education) (**Table 4**).

**Table 4:** Multivariate analysis on interviewees who did not know the Portuguese-language terms for stroke (“AVC” or “derrame”)

		Univariate		Multivariate	
		OR	95% CI	OR	95% CI
**Level of education**					
	Incomplete elementary school	5.88	3.61-9.57	6.10	3.73-9.96
	Completed elementary school	3.33	2.14-5.18	3.29	2.10-5.14
	Completed high school	1.50	1.03-2.17	1.51	1.04-2.20
	Completed tertiary education	1.00		1.00	
**Do you know anyone who has suffered a stroke?**					
	Yes	1.00		1.00	
	No	1.71	1.25-2.34	1.79	1.28-2.48
**Gender**					
	Female	1.00		–	
	Male	1.23	0.93-1.63		
**Age**					
	20-59 years	1.00		–	
	60 years and over	0.74	0.56-0.99		

OR = odds ratio; CI = confidence interval.

Knowing someone who had had a stroke reduced the chances of not knowing the terms relating to the disease (odds ratio, OR = 0.56; 95% CI 0.4-0.78). Among those who knew someone who had had a stroke, there were no statistically significant differences regarding sex, age or education level.

## DISCUSSION

Because of the impact of vascular diseases in Brazil and around the world, especially stroke, interventions involving education and preventive measures should be encouraged. Improving the population's knowledge about this subject can positively impact the mortality and complication rates associated with this disease.^5^

As a potentially treatable condition, albeit with a limited therapeutic window, the prognosis for stroke depends on the population's education and knowledge levels. Better education and knowledge enables people to quickly recognize a stroke episode and call the emergency service.^2^^,^^4^^,^^5^ In this context, medical entities have been investing in educational programs with the aim of achieving better results with regard to controlling cardiovascular and cerebrovascular diseases.^6^^–^^8^

This study revealed alarming data regarding the population's knowledge on preventing, recognizing and reacting to stroke. Although the sample studied had a significantly higher education level than the average for the state of São Paulo, its knowledge of stroke was unsatisfactory.

Knowledge of stroke directly correlates with the level of education, as already observed in studies conducted in developed countries.^9^ The same correlation was found in the present study (60.8% among the individuals with the highest education level versus 22% among those with the lowest level). Those in the subgroup with the highest education level were better able to recognize the signs and symptoms (19.3% versus 9.9%), risk factors (42.2% versus 19.8%) and preventive measures (34.6% versus 15.4%). This group was also better aware of the immediate steps to be taken when a stroke episode is recognized, such as seeking an emergency service (64.5% versus 38.6%).

These data contrast with what was found in a previous study,^10^ in which, irrespective of the level of education, 70% would call an emergency service, whereas in the present sample, only 57.8% would do so. Our study showed that only 2.9% of all the participants knew the importance of taking note of the timing of the onset of symptoms. Among those who know someone who had had a stroke, 44.31% and 31.78% did not know how to define stroke or list the appropriate measures to be taken if faced with a stroke episode, respectively.

The above scenario may reflect a lack of investment by the government in policies to educate the population and provide training in recognizing medical emergencies. In a systematic review, Saver reported that delayed access to emergency services was directly related to neuronal loss.^11^ In addition, rapid access to emergency services correlates with the likelihood of better treatment, such as thrombolysis. In a meta-analysis also published by Saver in 2016, the efficacy of administering endovascular therapy within seven hours of the onset of symptoms became evident.^12^ The 2018 guidelines of the American Heart Association (AHA) support the recommendation of thrombectomy for patients eligible for the procedure, for up to 24 hours after stroke onset in selected cases; the prognosis is better when the procedure is performed early.^13^ Investing in minimizing the delay in seeking an emergency service would substantially reduce morbidity, mortality and stroke-related costs.

A Brazilian study by Pontes-Neto et al.,^8^ with 814 participants who were interviewed in five major cities including São Paulo in 2007, generated results concerning the lack of information. One of its findings was that less than half of the population studied (38.7%) would seek an emergency service in the event of a stroke episode. In a study carried out by the team from São Paulo during a stroke campaign in 2011, similar data were found: less than half of the participants reported that they would seek an emergency service (33.6%).^6^ In contrast, more than half of the people interviewed in a study conducted in Belo Horizonte in 2014 said that they would take this measure (66.8%).^14^ In the present study in 2016, more than half of the interviewees said that they would react by seeking an emergency service (57.8%). These data suggest that a progressive improvement in the level of knowledge about this subject may have occurred.

Regarding preventive measures, the most frequent response was that obesity needed to be controlled (48.51%), whereas only 27.5% mentioned controlling high blood pressure as an important measure. According to the AHA, there is a consensus across multiple meta-analyses that controlling high blood pressure is the most beneficial measure for reducing the risk of stroke.^15^ The results from the present study contrast with findings reported in other studies, in which high blood pressure was referred to as the main risk factor: Nordhorn et al.^7^ in Berlin (43%) and a study previously conducted in São Paulo in 2011 (41.8%).^6^ There is a need to raise the awareness of the population about the impact of controlling high blood pressure upon prevention of cerebrovascular events.^14^

Thus, there is a need to disseminate information on stroke to the general population. This information remains scarce across all levels of education, especially regarding preventive measures. There is a notable disparity in the knowledge about the disease, as became evident from our multivariate analysis, with a greater lack of knowledge in the less educated population, which is precisely the population with the highest incidence of the disease.

## LIMITATIONS

The main limitation of this study related to its use of a convenience sample. This failed to represent the population of the state of São Paulo. The same limitation was observed in other studies conducted on this same subject. There was a selection bias in the present study, as shown in **Table 1**, since the interviewees’ mean age and level of education differed from the state averages. In our sample, the population was older than the state average, although it was younger than the population at greatest risk of stroke. This is not necessarily a limitation, since the concept of stroke prevention should be taught to the entire population, irrespective of age.

Because the population studied was composed of people with a higher level of education than the general population, our results may have underestimated the lack of knowledge about the subject. In addition, the symptoms mentioned by the participants were not specific to stroke. Furthermore, correct responses do not necessarily mean that the participants would know how to identify a stroke.

Another limitation was the lack of information on the number of people who refused to respond to the questionnaire. It is possible that the participants who agreed to respond were ones who were most interested in this matter and, accordingly, already had some knowledge of the subject.

The source of knowledge among these passers-by was not evaluated. This can be considered to be a limitation, since this could have provided an opportunity to understand what types of campaigns in the media would have the best reach regarding this disease.

## CONCLUSIONS

Despite the severity and prevalence of stroke, the population still has little information on this disease. In this context, the importance of mounting campaigns to improve prevention and treatment of stroke and contribute to healthcare policies becomes evident.
